# IgG4-related disease as multiple head and neck swellings: supported by histopathology and immunohistochemistry

**DOI:** 10.22336/rjo.2022.32

**Published:** 2022

**Authors:** Obaidur Rehman, Dipankar Das, Kasturi Bhattacharjee, Vatsalya Venkatraman, Harsha Bhattacharjee, Apurba Deka

**Affiliations:** * Department of Ocular Pathology, Sri Sankaradeva Nethralaya, Guwahati, Assam, India; ** Department of Oculoplasty and Oncology, Sri Sankaradeva Nethralaya, Guwahati, Assam, India; *** Department of Comprehensive Ophthalmology, Sri Sankaradeva Nethralaya, Guwahati, Assam, India

**Keywords:** IgG4-RD, immunohistochemistry, histopathology, plasma cells, IgG4

## Abstract

A 66-year-old North-East Indian male presented with bilateral eyelid swelling, ptosis, and bilateral submandibular gland enlargement. Dry skin on both arms was another peculiar complaint. Contrast enhanced CT scans revealed homogenously enhancing, diffusely enlarged lacrimal glands and blood investigations showed raised serum IgG4 levels. Histopathology from lacrimal gland biopsy showed lymphoplasmacytic infiltrates in storiform pattern. Immunohistochemistry showed 35% plasma cells positive for IgG4. A diagnosis of IgG4-related disease was made, due to supportive histopathology, immunohistochemistry, and serum IgG4 levels. The patient showed excellent response to systemic immunomodulators.

**Abbreviations:** IgG4-RD = IgG4-related disease, CECT = Contrast-enhanced computed tomography, ACE = Angiotensin converting enzyme, IHC = Immunohistochemistry, HPF = high power field, IgG4-ROD = IgG4-related ophthalmic disease, ACR/ EULAR = American College of Rheumatology/ European League Against Rheumatism

## Introduction

IgG4-related disease (IgG4-RD) is a multi-system inflammatory disorder that has gained acknowledgement in recent years and is characterized by high serum IgG4 levels along with plasma cells positive for IgG4 on histopathology [**1**]. Ophthalmic presentations of IgG4-RD in various forms of orbital and adnexal inflammation have been described in literature [**2**]. A unique case of intraocular inflammation in IgG4-RD, which presented as a masquerade of ciliary body melanoma has previously been reported from North-East India [**3**].

We report a case of a 66-year-old North-East Indian male, who presented with multiple, painless swellings in the head and neck region, including swelling of bilateral lacrimal glands and submandibular glands. Immunohistopathology was characteristic of IgG4-RD and the patient was managed accordingly.

## Case report

**Fig. 1:**
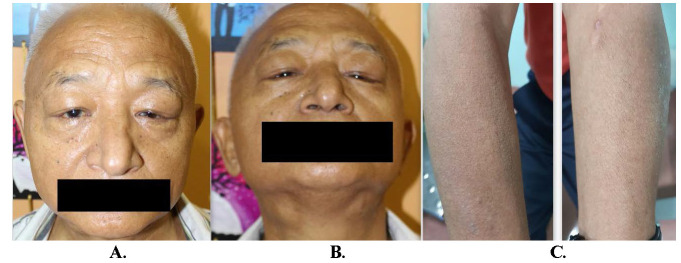
**A.** Bilateral lacrimal gland; **B.** Visible bilateral submandibular gland swellings; **C.** Dry skin on the arms

A 66-year-old North-East Indian male presented to the outpatient department with complaints of swelling and drooping of both his eyelids along with swellings on both sides of the neck for the past seven months. The swellings were painless in nature and had progressively enlarged over time. External examination revealed superolateral swellings in both eyelids in the lacrimal gland region, having a firm and rubbery consistency (**Fig. 1A**). Visible swellings were also noted in bilateral submandibular glands area (**Fig. 1B**) while mild enlargement of both parotid glands could also be palpated. The enlarged salivary glands had the same consistency as the lacrimal glands. A peculiar note of dry skin was also made on both his arms (**Fig. 1C**). Ocular examination was unremarkable on both sides, including the anterior and posterior segments.

**Fig. 2:**
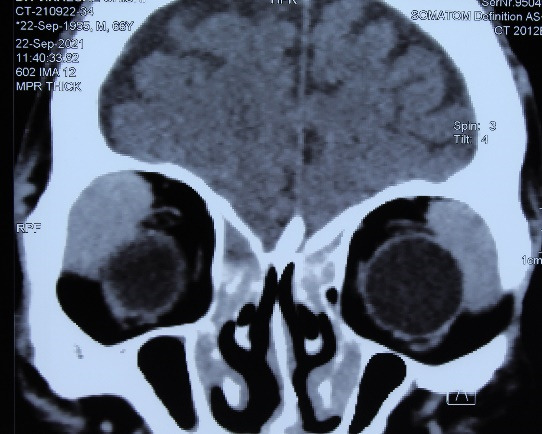
CECT scan in the coronal view showing diffusely enlarged lacrimal glands with homogenous enhancement

**Fig. 3:**
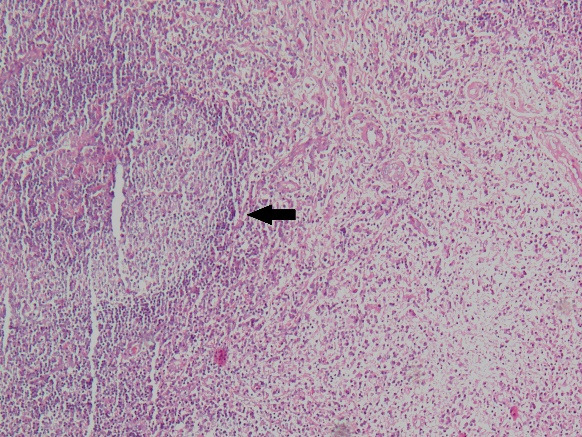
Hematoxylin and Eosin-stained slide showing dense lymphoplasmacytic cells with germinal center (marked with black arrow). Some areas have storiform pattern and obliterative vasculitis (x 10X)

Past medical history revealed a previous fine needle aspiration cytology examination from the enlarged submandibular lymph node showing benign lymphoid hyperplasia. The patient was advised to undergo contrast-enhanced computed tomography (CECT) scans of the orbits, which revealed diffusely enlarged lacrimal glands with homogenous enhancement in the coronal view (**Fig. 2**). Differential diagnoses of Mikulicz syndrome, Sjogren syndrome and lacrimal gland lymphoma were kept in mind, and the patient was planned for lacrimal gland biopsy from the left side with histopathological examination. Intra-operatively, diffuse involvement of surrounding orbital tissues and muscles was noted. Histopathology showed diffuse sheets of plasma-lymphoid cells with germinal centers (**Fig. 3**). Cells in different stages of maturation were noted but no emperipolesis was observed.

**Fig. 4:**
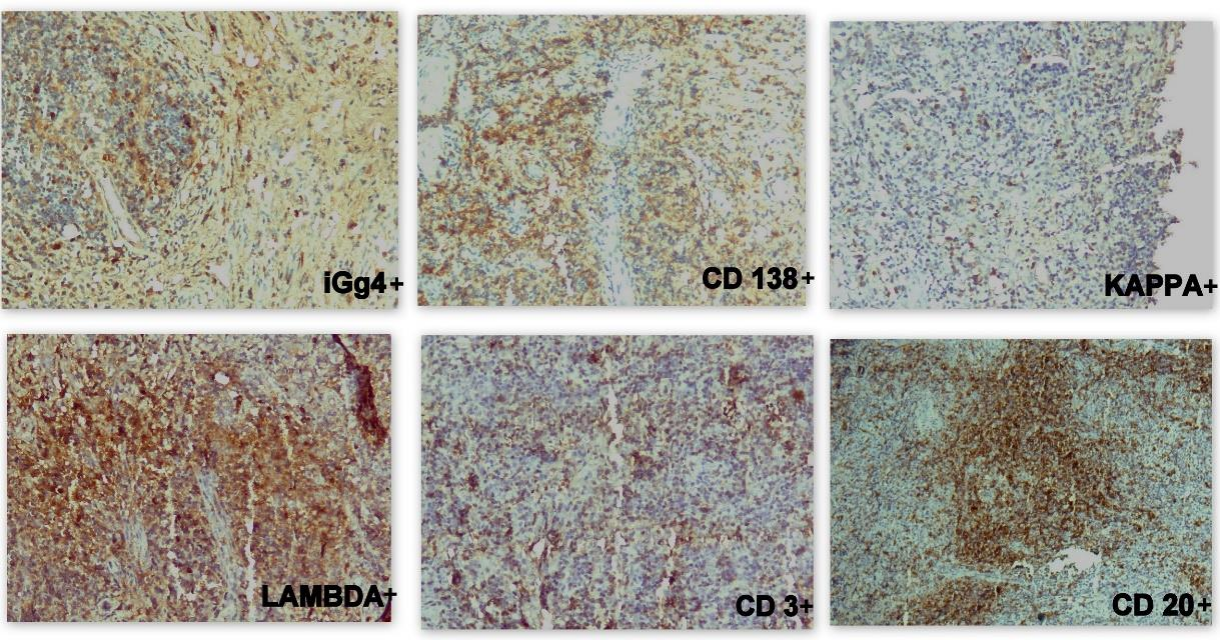
IHC showing positivity for different markers (x 10X)

Meanwhile, the patient was also asked to look for a rheumatology opinion. Thorough evaluation including whole body imaging and a series of blood investigations was performed. The patient was negative for RA factor, Anti SS-A (Rho) antibody, Anti SS-B (La) antibody and Anti-CCP, but excessively raised serum IgG4 levels (80 grams/ liter) were a notable finding. Serum Angiotensin converting enzyme (ACE) levels were also high but a high-resolution CT scan ruled out sarcoidosis as a diagnosis. IgG4-RD was anticipated and further, immunohistochemistry (IHC) was performed. IHC (Scytek, Logan, Utah, USA) was positive for CD20, CD3, kappa, lambda, CD138, along with 35% positivity in plasma cells for IgG4 (**Fig. 4**). Slides were compared with positive and negative controls. 35 IgG4-positive cells per high power field (HPF) were noted.

Due to high serum IgG4 levels and tissue positivity for IgG4, a final diagnosis of IgG4-RD was made. Consulting with the rheumatologist, the patient was started on a tapering dose of oral prednisolone and tablet Mycophenolate mofetil, according to weight. On subsequent follow-up at 2 weeks, the multiple swellings had drastically reduced in size. The patient is still under close follow-up. 

## Discussion

IgG4-RD was described for the first time nearly two decades ago and since then has been known to cause fibroinflammatory mass lesions in almost any body organ [**4**]. This disease strongly affects few organs that include orbital tissues, lacrimal glands, salivary glands, kidneys, retroperitoneum, etc. [**5**]. IgG4-related ophthalmic disease (IgG4-ROD) has been reported to involve the sclera, soft tissues of orbit, choroid, lacrimal system, and extraocular muscles [**1**]. IgG4-RD with systemic involvement occurs mostly in middle to old age (median age: 58 to 67 years), while a slightly younger average age (56.7 years) has been noted in men with IgG4-ROD [**1**].

Involvement of submandibular and lacrimal glands is common in the head and neck region [**6**]. The enlargement of these glands has been described to be painless and progressive with elastic consistency, similar to what was seen in our case. In IgG4-RD involving head and neck, submandibular gland enlargement is more commonly noted, in contrast to predominant parotid enlargement in Sjogren’s syndrome [**6**].

The American College of Rheumatology/ European League Against Rheumatism (ACR/ EULAR) committee has recently proposed new criteria for classification of IgG4-RD [**7**]. A multi-disciplinary team of specialists has laid down 3-step criteria, the first step being the demonstration of a possible IgG4-RD involvement in one of the possible 11 organs, the second step being the screening through 32 exclusion criteria and the third step involving 8 weighted domains that have the inclusion criteria. Lastly, a sum of all inclusion criteria points was done. The validation cohort of this study showed that a score of 20 carried 99.2% specificity for IgG4-RD diagnosis. In our case, the total of the inclusion criteria points came out to be 29. 

Histopathology showed dense plasma-lymphocytic proliferation in storiform pattern within sheets of lymphoid infiltration along with obliterative phlebitis in the tissue. IHC revealed polyclonality in CD3 and CD20 positivity, thus ruling out a malignant lesion. CD138 immunostaining was positive for plasma cells, but a polyclonal expression of kappa and lambda signified absence of plasmacytoma. IgG4 immunostaining was positive for 35% plasma cells, with 35 IgG4-positive cells seen per HPF. 

There is no current report of IgG4-RD presentation with dry eye and dry skin. Since lacrimal glands are involved, a possibility of dry eye exists in these patients. Dry skin was a peculiar finding in our patient and could be a unique presentation of IgG4-RD.

Response to systemic steroids in IgG4-RD is high initially but it has been suggested that only a limited time period exists for efficiency [**1**]. Immunomodulators can be used in conjunction with a tapering dose of steroids or as steroid-sparing agents.

Previous literature has shown that IgG4-ROD is a close mimicker of malignancy, particularly lymphoma [**8**-**10**]. IgG4-RD may rarely convert into a low-grade lymphoma; thus, these patients require a close follow-up and monitoring.

## Conclusion

IgG4-related disease can present with swelling of glands of the head and neck. Diagnosis requires a high degree of clinical suspicion. Clinicians must be aware of this clinical entity and its various presentations, to diagnose it early and tackle it effectively.

**Conflict of Interest Statement**

The authors state no conflict of interest. 

**Informed Consent and Human and Animal Rights statement**

Any information that could contribute to identify the patients, including the patients’ names, initials or hospital numbers have not been published in written descriptions or photographs. The manuscript was shown to the patient before submission to the journal and an explicit informed consent was obtained for the purpose of publication. 

**Authorization for the use of human subjects**

Ethical approval: The research related to human use complies with all the relevant national regulations, institutional policies, it is in accordance with the tenets of the Helsinki Declaration and has been approved by the review board of Department of Ocular Pathology and Department of Oculoplasty and Oncology, Sri Sankaradeva Nethralaya, India. 

**Acknowledgement**

None. 

**Sources of Funding**

Nil. 

**Disclosures**

None. 

**Commercial relationships**

Nil.

**Competing interests**

None.
